# Odontalgia

**Published:** 1875-05

**Authors:** 


### Our Exchanges.

ODONTALGIA.—The police reporter of the *Atlanta Daily Herald* is, to use his own vernacular, a "festive" fellow, of infinite jest and humor. In a recent issue he brings to our table the following very expressive delineation of the animus of an odontalgia martyr:

"Gracious, Godfrey! How it pains me,
Lordy! don't that old tooth jump!
Seems as though ten thousand devils
Pried with crowbars around its stump.

Whew! can't some one give me something,
Just to stop this blasted pain?
Hot drops! laudanum! cloves, or hop-bag!
Quick! or I shall be insane.

Stop that tarnal baby's squalling!
Jethrew! don't my tooth ache sweet.
Darn that cat! I'd like to kill it,
Always under someone's feet.

Jove, I'd like to fight with some one,
Just to get my jaw stove in;
Fire! Murder! Godfrey diamonds!
Oh, it's aching now like sin.

Howling, am I? well I know it,
And I guess that you'd howl too,
If you had a blasted toothache,
Same as this, troubling you.

'Course I know it don't relieve me,
But I'm crazy with the pain,
Ain't there anything to ease it?
Let me try the hops again.

There now, gently, place them easy,
Phew! they're hot! just let em cool,
Well, put 'em on. You're bound to burn me;
There, you've done it! Darn a fool!"

